# Quad-phased data mining modeling for dementia diagnosis

**DOI:** 10.1186/s12911-017-0451-3

**Published:** 2017-05-18

**Authors:** Sunjoo Bang, Sangjoon Son, Hyunwoong Roh, Jihye Lee, Sungyun Bae, Kyungwon Lee, Changhyung Hong, Hyunjung Shin

**Affiliations:** 1 Department of Industrial Engineering, Ajou University, 206, World cup-ro, Yeongtong-gu, Suwon-si, Gyeonggi-do Republic of Korea; 2 Department of Psychiatry, Ajou University School of Medicine, 206, World cup-ro, Yeongtong-gu, Suwon-si, Gyeonggi-do Republic of Korea; 3 Department of Digital Media, Ajou University, 206, World cup-ro, Yeongtong-gu, Suwon-si, Gyeonggi-do Republic of Korea

**Keywords:** Data mining modeling, Variable selection, Support vector machine, Artificial neural network, Decision tree, Tree visualization, Dementia diagnosis, Computer-aided diagnosis (CAD)

## Abstract

**Background:**

The number of people with dementia is increasing along with people’s ageing trend worldwide. Therefore, there are various researches to improve a dementia diagnosis process in the field of computer-aided diagnosis (CAD) technology. The most significant issue is that the evaluation processes by physician which is based on medical information for patients and questionnaire from their guardians are time consuming, subjective and prone to error. This problem can be solved by an overall data mining modeling, which subsidizes an intuitive decision of clinicians.

**Methods:**

Therefore, in this paper we propose a quad-phased data mining modeling consisting of 4 modules. In Proposer Module, significant diagnostic criteria are selected that are effective for diagnostics. Then in Predictor Module, a model is constructed to predict and diagnose dementia based on a machine learning algorism. To help clinical physicians understand results of the predictive model better, in Descriptor Module, we interpret causes of diagnostics by profiling patient groups. Lastly, in Visualization Module, we provide visualization to effectively explore characteristics of patient groups.

**Results:**

The proposed model is applied for CREDOS study which contains clinical data collected from 37 university-affiliated hospitals in republic of Korea from year 2005 to 2013.

**Conclusions:**

This research is an intelligent system enabling intuitive collaboration between CAD system and physicians. And also, improved evaluation process is able to effectively reduce time and cost consuming for clinicians and patients.

**Electronic supplementary material:**

The online version of this article (doi:10.1186/s12911-017-0451-3) contains supplementary material, which is available to authorized users.

## Background

It is expected that population of worldwide senior citizens would increase from 900 million to 2 billion between 2015 and 2050. Among them, more than 20% are expected to suffer cognitive disorder including dementia and mild cognitive impairment and also mental disorder including depression and anxiety disorder [1]. Among such impairments, it is well clarified that if dementia can be early diagnosed and its intervention time based on medicine and non-medicine treatment is shortened, the progress of dementia is delayed [2–5]. In general, evaluation processes for dementia consist of very complicated and various additional examinations such as diagnostics of dementia types, measurement of its seriousness starting with screening patients with dementia through simple medical examination [6, 7]. However, diagnostic value and its clinical meaning against various medical examinations performed during diagnostic processes are questioned recently, moreover these multiple and complicated evaluation processes by physician are time consuming, subjective and prone to error [8].

Recently, Computer-Aided Diagnosis (CAD) is introduced in order to alleviate such skepticism for diagnosis of various disease. CAD is a research that applies clinical data to machine learning algorism to help physicians to examine patients and ultimately to automate clinical decisions [9]. Through previous studies, we are able to look into researches which utilized an excellent machine learning predictive model such as Support Vector Machine (SVM), Artificial Neural Network (ANN). Stefan proposed SVM model by which he classified causes of Alzheimer’s disease by fronto-temporal lobar degeneration (FTLD) [10]. Ramírez also proposed CAD study to enhance early detection of Alzheimer’s dementia using SVM [11]. And Chen applied various machine learning algorisms such as Discriminant analysis, Decision Tree (DT) and SVM in order to forecast Very mild dementia (VMD) [12]. In addition, there are various studies using ANN model to diagnose cancers [13–15].

**Fig. 1:**
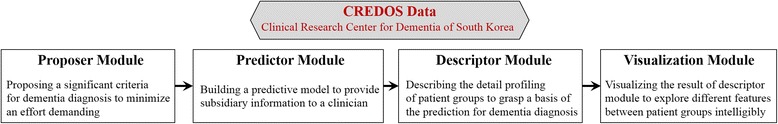
Schematic description on the procedure of the proposed method: Quad-phased data mining modeling for dementia diagnosis

In this study we propose a systematic and overall data mining modeling to improve dementia evaluation process. The proposed method consists of a total 4 modules: Proposer module, Predictor module, Descriptor module, and Visualization module. Figure 1 indicates whole structure for this study. Conventional dementia diagnostic models have consisted of too many examination and sometimes ones with discrepancy. Therefore, there are some difficulties that clinical physicians suffer to configure overall conditions actually for patients when they receive examination results on site. In order to alleviate this problem, in proposer module, significant diagnostic criteria for dementia are selected to improve an examination system. We proposed a *kScale* variable selection method looking into various variable selection methods in existing studies. With the results of previous module, in predictor module, a predictive model with machine learning algorithm is constructed. The model subsidizes an intuitive decision of clinicians for diagnosing dementia. The most excellent predictive model is selected by comparing three well-proven machine learning models: SVM, ANN and DT. Good performance of the predictive model is not enough in clinical areas. Detailed interpretation of results is more important so that clinical physicians can understand results of mathematical model better. Therefore in descriptor module, patients are segmented depending on characteristics and detailed profiles of each patient group to describe results of diagnostics are provided. Lastly in visualization module, effective visualization exploring characteristics of patient groups is provided. The proposed model is applied for CREDOS study which contains clinical data collected from 37 university-affiliated hospitals in republic of Korea from year 2005 to 2013.

## Methods

In following sections, more detailed explanation about Proposer, Predictor, Descriptor and Visualization Module are mentioned in their order which constitute the Quad-phased data mining modeling.

### The proposer module

In proposer module, important diagnostic criteria among various examination criteria to diagnose dementia are selected. This belongs to dimensionality reduction of variables in conventional data pre-processes [16]. There are two approaches to scale down dimensionality of variables: Variable selection method that maintains intrinsic meaning of variables and variable extraction method that extracts meaning from whole variables by combining them [17]. Since it is important to interpret results of predicted diagnostics using intrinsic meaning of diagnostic criteria, in this study variable selection methods are more suitable [18–20]. In general one of the methods is chosen. Although one of the methods is chosen in general, it is difficult to recognize which method plays the most crucial for variable selection since results of variable selection from each method. Therefore, we propose ‘kScale variable selection method’ that accounts for flexibility by examining different results from several methods. The procedure is given as the following. First, we independently apply k different variable selection methods to the original data. Then, we assign importance to each variable by counting the frequencies of selection from each method. Given M variables x_m_ (m = 1 … M), importance of variables is calculated from *k* number of variable selection methods as following.

$$ kScale\left({x}_m\right)={\displaystyle \sum_{k=1}^K} S C(k), $$

$$ S C(k)=\left\{\begin{array}{l}1,\kern0.5em  if\ {x}_m\  satisfies\  selection\  criterion\hfill \\ {}0,\kern0.5em  otherwise\hfill \end{array}\right. $$

In this case, *SC*(*k*) is assigned to be one when selection criterion for *k* ^*th*^ variable selection method is satisfied. Therefore, the bigger *kScale* indicates that its variable is more important representing well-matched opinions among several variable selection methods. In this study, we used Chi-square test, Decision tree, Logistic Regression described in Table 1.

**Table 1:** Variable selection methods to be used

Variable Selection Method		Definition	Selection Criterion
Chi-square Test (univariate)		${\chi}^2={\displaystyle \sum_j}\frac{{\left({O}_j-{E}_j\right)}^2}{E_j}$ O_j_ is the observed frequency and E_j_ is the expected frequency of class j	*p value* < 0.05
Decision Tree	CHAID	(*based on Chi* − *square Test*)	*Importance* > 0.001
	CART	$$ \begin{array}{l} Entropy(t)=-{\displaystyle \sum_j} p\left( j\Big| t\right) \log p\left( j\Big| t\right)\hfill \\ {} GAI{N}_{split}= Entropy(p)-\left({\displaystyle \sum_{i=1}^k}\frac{n_i}{n} Entropy(i)\right)\hfill \end{array} $$	*Importance* > 0.001
	C4.5	$$ \begin{array}{l}\begin{array}{l} GIN I(t)=1-{\displaystyle \sum_j}{\left[ p\left( j\Big| t\right)\right]}^2\hfill \\ {} p\left( j\Big| t\right)\ is\ t he\ relative\ frequency\ of\ class\ j\ at\ node\ t\hfill \end{array}\\ {} GIN{I}_{split}={\displaystyle \sum_{i=1}^k}\frac{n_i}{n} GIN I(i)\end{array} $$	*Importance* > 0.001
Logistic Regression	LR (1)	$F(x)=\frac{1}{1+ exp\left({\beta}_0+{\beta}_1{x}_1 \dots {\beta}_n{x}_n\right)}$	*p value* < 0.05
	LR (1)		*p value* < 0.01

* Note that the importance in selection criterion in Decision Tree is different from the aforementioned ‘importance’. The former is simply the weights imposed on a largely contributing variable for classification of sample with growth of the tree

### The predictor module

**Fig. 2:**
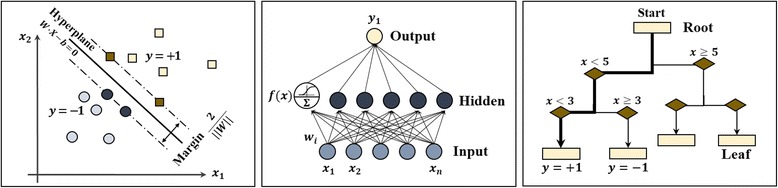
Predictive models: **a** SVM, **b** ANN, and **c** DT

In predictor module, a predictive model to determine whether or not a patient is under dementia are constructed and this is for helping clinical physicians in diagnosis. This model uses variables extracted from Proposer Module as input variables, and Clinical Dementia Rating (CDR) variable as target variable by making binary for ‘normal or dementia’. In this study we use SVM, ANN and DT among machine learning algorisms which are described in Fig. 2. And also AUC which stands for area under a receiver operating characteristic (ROC) curve is used to evaluate predicted performance for the three models. AUC is an threshold-independent index to evaluate performance of predicted model [21]. The remainder of this section mentions brief explanation for SVM, ANN, and DT with important points in this study.

#### Support vector machine

SVM is supervised learning model which can be used for classification and regression analysis [8, 11, 22]. In this problem, patients will be divided into two states of diagnosis ‘normal or dementia’. A technical definition that x and y refer to patient records and state of diagnosis respectively is commonly applied to ANN and DT. A conceptual description of SVM is shown in Fig. 2a. Given a training dataset of n, $\left(\overrightarrow{{\mathrm{x}}_1},{y}_1\right),\dots, \left(\overrightarrow{{\mathrm{x}}_{\mathrm{n}}},{y}_n\right)$, the 2-dimensional vector points $\overrightarrow{{\mathrm{x}}_{\mathrm{n}}}$ can be divided into two classes y_i_ which are either +1 or -1. We call the nearest training-data point of any class as a functional margin. SVM has a goal of finding the optimal decision boundary written as $\mathrm{f}\left(\mathrm{x}\right)=\overrightarrow{\mathrm{w}}\cdot \overrightarrow{\mathrm{x}}-\mathrm{b}=0$ which maximizes the margin. Samples on the margin are called the support vectors. If the problem is not linearly separable, we can write the optimization problem as follows

$$ \mathrm{minimize}\ \frac{1}{\mathrm{n}}{\displaystyle \sum_{i=1}^n}{\xi}_i+\uplambda {\left|\left|\mathrm{w}\right|\right|}^2, $$

$$ \mathrm{s}.\mathrm{t}.\kern0.75em {\mathrm{y}}_{\mathrm{i}}\left(\overrightarrow{\mathrm{w}}\cdot \overrightarrow{\mathrm{x}}+ b\right)\ge 1-{\xi}_i, $$

$$ {\xi}_i\ge 0,\kern0.5em \mathrm{i}=1,\dots, \mathrm{n}. $$

The parameter *ξ*_*i*_ is the non-negative slack variable and the parameter λ determines the tradeoff between increasing the margin-size. Please see the references for more details about SVM [23, 24].

#### Artificial neural network

ANN is also a well-known analytical system for being applied to outcome prediction of diseases [13, 14]. ANN is inspired by the concept of humans’ central nervous systems. As described in the Fig. 2 b, nodes which represents ‘neuron’ are connected together to form a network and comprises three types of layers: the input layer (I), the hidden layer (H) and output layer (O). In this problem, the nodes in the input layer supply input patient records to the nodes in the hidden layer via weighted connections. Then, the nodes in the output layer represents the result of diagnosis ‘normal or dementia’ by a weighted sum of the signals from the hidden nodes on the basis of a transfer function. Mathematically, *g*_*i*_^*I*^(*x*) becomes activation functions from input layer and *g*_*i*_^*I*^(*x*) becomes that from hidden layer. We can write the overall a neuron’s network function *f*(*x*) which is a weighted sum of the signals from the hidden nodes as follow

$$ f(x)={g}^o\left({\displaystyle \sum_i^{n_h}}{w}_i{g}_i^h(x)\right), $$

where *g* ^*o*^ refers to the predefined or activation function in output layer.

A set of weights *w*_*i*_ is determined by training ANN model with data. In ANN, the accuracy of the model often depends on the structure, i.e. the number hidden nodes, and the initial weights associated with the connections between the nodes. Generally, the number of hidden nodes is selected by trial-and-error fashion and the initial weights are randomly chosen. Please see the references for more details about ANN [25, 26].

#### Decision tree

The decision tree is a decision support system with the form of graph or flowchart. A briefed concept is shown in Fig. 2 c DT determines important variables to classify dataset in tree configuration and their threshold values [27]. Along with the way to determine the split, three different types of decision tree are introduced in Table 1 which are used for variable selection (CHAID, CART and C4.5) in the proposer module. However, we used CHAID (chi-squared Automatic Interaction Detection) as a predictive model which is based on chi-square test splitting rule for nominal target criterion. Please see Table 1 and references for more details about DT [28–30].

### The descriptor module

In descriptor module, we provide aspect of descriptions based on input and output values of a predictive model which is finally selected from predictor module. In other words, this is to identify why a patient is classified to a dementia from the predictive model. For this analysis, data mining method, which combines the predictive model with additional description model, can be used [31]. In this study, we additionally configure DT as an interpretable model. This enables to identify characteristics of data segmented to leaf group based on important variables from tree and their thresholds. In other words, this makes profiles for patient groups with ‘normal or dementia’ using diagnostic criteria and their evaluation results. As mentioned earlier from predictor module, DT is enough to be used for a predictive model. If predicting performance of DT is better that those of SVM and ANN, both prediction and description are fulfilled only by DT. However, if predicting performance of SVM or ANN is better than DT, DT is additionally configured for helping clinicians understand predicted results better. It is possible by using predicted target values obtained from SVM or ANN together with the training samples as input to DT. It means that DT re-track predicted results not newly predict.

### The visualization module

In visualization module, useful visualization is provided to explore characteristics of patients who are grouped in descriptor module. It is conducted by way of maximizing visual effects of DT. Basically, contrasting colors for patients with normal or dementia and different diagnostic criteria are able to distinguish them immediately. And showing up unique path which is through important criteria, individual analysis profiling patient groups is possible. Also, as thickness of each path is proportional to the number of patients, figuring out a characteristic of patients included in a certain group is instinctive.

## Experiments

### Data

In order to verify proposed method, we used clinical data called ‘CREDOS’. CREDOS study registered on ClinicalTrials.gov (identifier: NCT01198093) recruited participants from 2005.1 to 2013.5 from 37 university-affiliated hospitals who were diagnosed with normal cognition, subjective memory impairment, mild cognitive impairment, vascular cognitive impairment, subcortical ischemic vascular dementia, Alzheimer’s disease, or other type of dementia by neurologist or psychiatrist. CREDOS study included 21,094 clinical and neuropsychological evaluation results from 14,917 participants. A more detailed description of CREDOS study has been published previously [32, 33]. In brief, CREDOS dataset comprised of demographic and baseline characteristics, a lot of information from caregiver and patient (Table 2). This information dataset included 14 diagnosis criteria which cover 486 subspecialized criteria. For CREDOS study, we excluded those who met the following criteria: (1) history of significant hearing or visual impairment rendering participation in the interview difficult; (2) history of following neurologic disorder (brain tumor, subarachnoid hemorrhage, epilepsy, encephalitis and metabolic encephalopathy) or other neurologic conditions that could interfere with the study; (3) history of psychiatric disorder including mental retardation, schizophrenia and bipolar disorder or other psychiatric conditions that could interfere with the study; (4) history of psychoactive substances other than alcohol; (5) history of physical illnesses or disorders including cancer, renal failure, hepatic failure, severe asthma or chronic obstructive pulmonary disease or other physical conditions that could interfere with the study. CREDOS study was approved by the institutional review board of the participating centers. All participants signed informed written consents. This study was approved by the institutional review boards at the clinical sites.

**Table 2:** Description of CREDOS dataset

Data	Description
Demographic and baseline characteristics	Age, gender, education
Information from Caregiver	Basic Activity of Daily Living (BADL), Caregiver-Administered Neuropsychiatric Inventory (CGA-NPI), Korean Dementia Screening Seoul-Instrumental Activities of Daily Living (S-IADL) Questionnaire (KDSQ),
Information from Patient	Clinical Dementia Rating (CDR), Global Deterioration Scale (GDS), Korean Mini-Mental State Examination (K-MMSE), Korean Version of Short Form Seoul Neuropsychology Screening Battery (SNSB) Geriatric Depression Scale (SGDS-K),

First of all, we screened 486 examination criteria in CREDOS dataset to 366 criteria. Then applied them to the kScale variable selection method. To build SVM, ANN and DT in predictor module, one of the criteria ‘CDR binary’ (normal or dementia) was set as target variable. And the experiment was conducted by dividing data set into 40% for training, 30% for test and 30% for validation.

## Results and Discussion

### Results of proposer & predictor modules

**Fig. 3:**
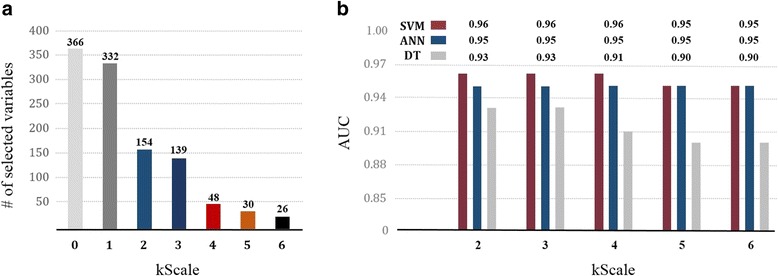
The result of proposer module and predictor module: (**a**) The number of selected variables by the *kScale*, and (**b**) The result of AUC

We applied the proposed *kScale* variable selection method to screened 366 criteria. Figure 3 a shows the number of variables that are extracted depending on *kScale*. To decide the number of variables among the results, we reflected the opinion of clinical physicians. As a result, 48 variables which were selected 4 times out of 6 variable selection methods were verified to be critical criteria that divide patients with normal and dementia.

In predictor module, SVM, ANN and DT have built and Fig. 3b indicates a graph which compared AUC results for each model according to the number of input variables. Its detailed values are shown in Fig. 3. When the number of input variables is 48, AUC has value of SVM = 0.96, ANN = 0.95, DT = 0.91 for the validation set. SVM has the biggest AUC value, thus SVM outperforms ANN and DT. Therefore, SVM was selected the best dementia predictive model. In terms of performance validation, the selected SVM achieved accuracy of 0.90 and precision of 0.85. In addition, Fig. 3b indicates that a case of using 48 variables is more effective than other cases. It is because, first of all if it is compared to a case that uses more variables, predicting performance is similar. Therefore, it is found that this is able to save time and cost 10 times more compared to a case that uses initial 480 variables. Also, since use of too less variables deteriorates predicting performance gradually, we are able to bring to a conclusion that use of 48 variables is suitable to maintain higher predicting performance.

**Fig. 4:**
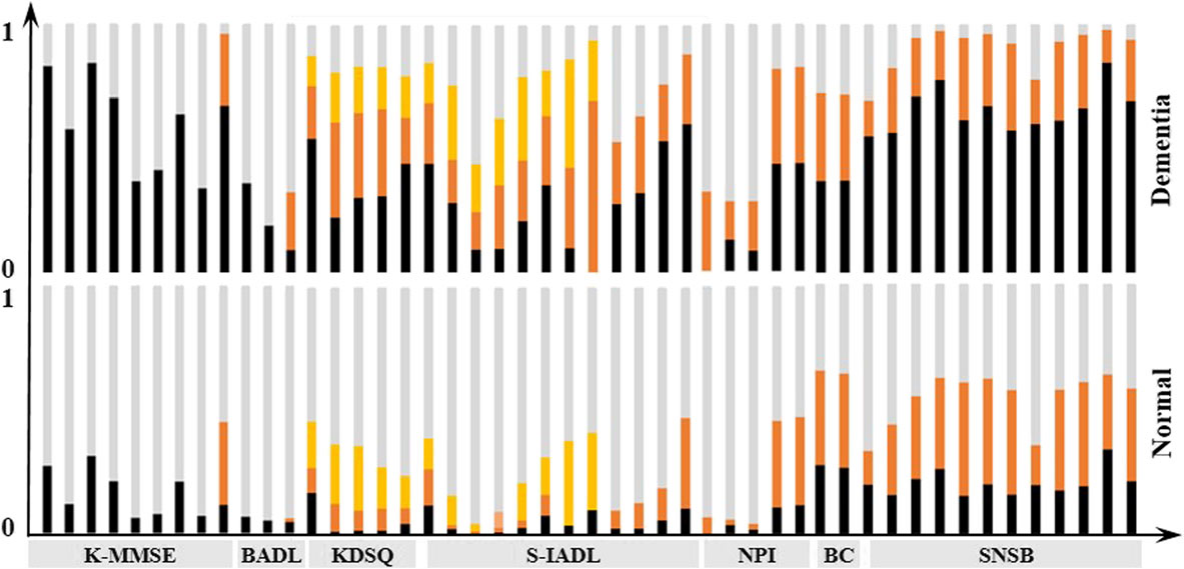
Value trend of selected variables

Figure 4 shows a graph indicating separate normalized number of patients by 48 selected examination criteria for patients diagnosed ‘dementia’ and ‘normal’. Darker colored part of each bar indicates patients who show signs of having dementia in each criteria. And the number of color in each bar is different depending on the each examination criteria’s scale. For example, K-MMSE has 2 (wrong, right) nominal, S-IADL has 4 (very strong, strong, weak, none) ordinal values, and SNSB has continuous values which are leveraged to three scale, etc. Comparing proportion of darker part of dementia (upper graph) to that of normal (lower graph), the proportion of dementia is larger in every bars. Therefore, finally selected 48 examination criteria are believed to be very useful variables to identify whether a patient is normal or dementia.

### Results of descriptor & visualization modules

**Fig. 5:**
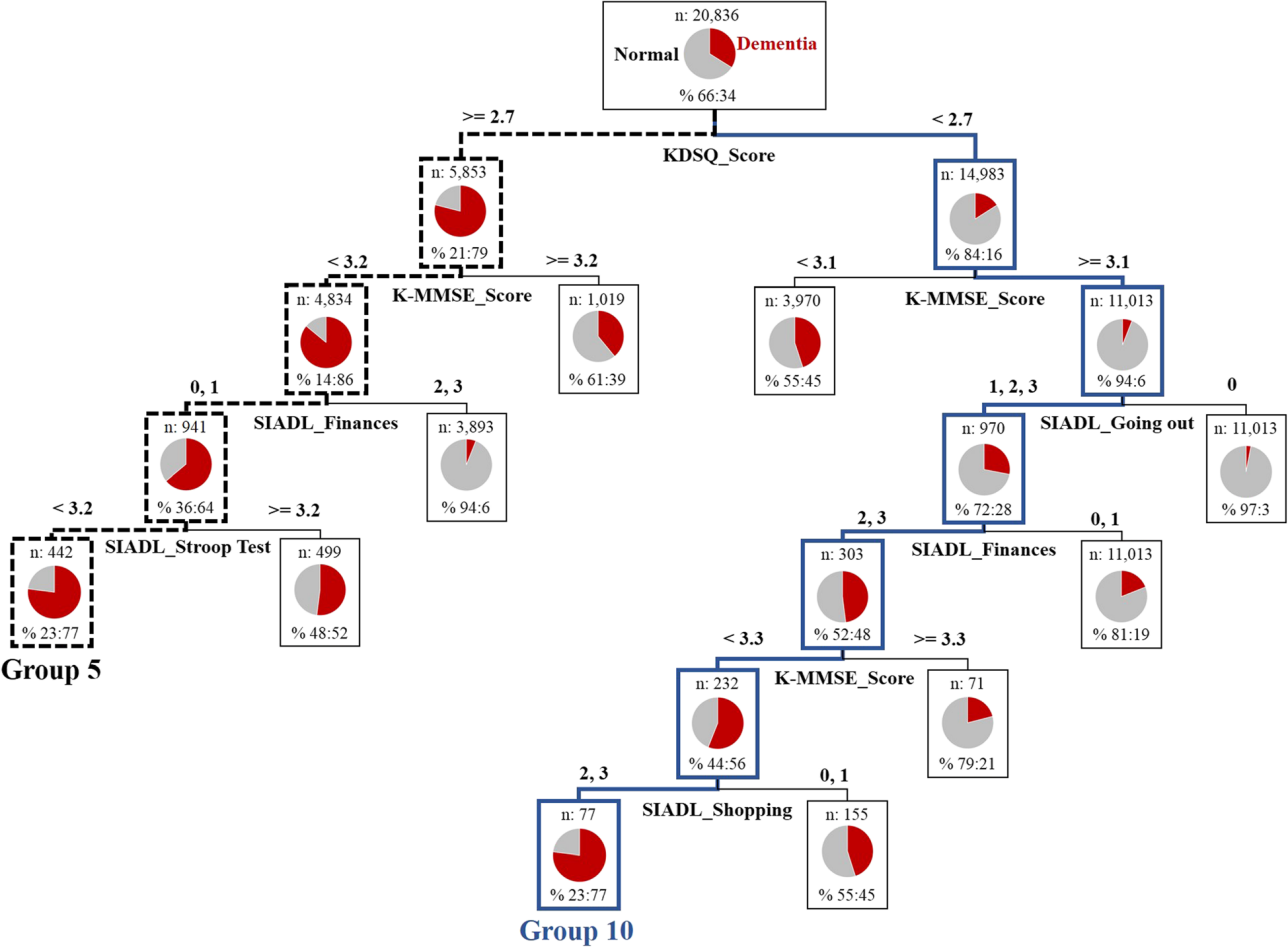
The result of profiling: group 5, group 10

Since SVM was selected for a final predictive model, an additional descriptive model is needed. Therefore, DT was configured using input value and predicted value of SVM. As a result, 20,836 clinical records are classified as 31 groups in leaf nodes. Each group can be profiled with importance of variables and threshold value which are determined as tree is growing. Figure 5 shows paths of group 5 and group 10 which are regarded as clinically meaningful and provides detailed profiling for each group below. The full list of 31 groups are provided in Table B in supplementary material (Additional file 1). And also accessible in [34].

To summarize some major diagnostic criteria, KDSQ that is an initial dementia identifying examination is a questionnaire to measure orientation, social cognition and mobility, and its higher score indicate that a patient is more likely to be dementia. And K-MMSE consists of mainly time orientation and place orientation, and its higher score means that a patient is more likely to suffer dementia. SIADL is an index to evaluate instrumental activity of daily living, which consists of questionnaire and its answers with a range from 0 to 3 for detailed criteria and as the score approaches 3, this means that a patient is more dependent of his or her care giver.

Group 5 includes people who have disorder worse than medium level, it is due to high score of KDSQ and low score of K-MMSE. In addition, they might have disorder of executive function and selective attention since low score of SIADL stroop test is found at the bottom of the tree. However, SIADL related to managing money indicated this group is proved to make an independent decision. Therefore, patients in this group, although it is assumed that their cognitive skills are deteriorated and such aggravation began to affect daily life, are more likely to live comparably independent life.

Group 10 includes people who are proved to be relatively under better condition as it marked low score of KDSQ and high score of K-MMSE. However, this group is believed to be hard to live independent life as people in this group marked high score in several criteria of SISDL in connection with going out nearby house, managing money and purchasing goods. Therefore, patients in this group, although they showed some deterioration in cognitive skill, are believed to have serious disorder especially for their daily life.

Table 3 shows results of profiling for two normal groups and 8 dementia groups among a total of 31 groups. Since each groups’ portion of dementia patients are more than 70%, particular distinguishing features are figured out.

**Table 3:** Profiling for 2 normal groups and 8 dementia groups whose portion of dementia patients are more than 70%

group	Profiling for segmented groups		Variables contributed to set the group profile	# of patients in the group (% of dementia patients)
1	Normal	A group with no deterioration of cognitive skills or with very weak symptom	KDSQ_Score (normal), K-MMSE_Score (normal), SIADL_Going out (normal)	10,043 (4%)
2		A group without any big difficulty to live daily life although patients in this group sometimes show a weak disorder of cognitive skill	Although patients in this group are diagnosed with dementia for some diagnosis, they are finally proved to be normal	2,905 (18%)
3	Deterioration of cognitive skills, facing difficulties in daily life	Dementia group with symptom more than medium level	KDSQ_Score (dementia), K-MMSE_Score (dementia), SIADL_Finances (dementia), SIADL_Cooking (dementia)	3,623 (93%)
4		A group with disorder in language skill and ideational apraxia	KDSQ_Score (dementia), K-MMSE_Score (dementia), SIADL_Finances (dementia), SIADL_Cooking (normal), SNSB_Praxis Ideomotor (dementia)	124 (88%)
5		A group in which patients are relatively likely to live independent life	KDSQ_Score (dementia), K-MMSE_Score (dementia), SIADL_Finances (normal), SNSB_Stroop Test (dementia)	442 (77%)
6		Initial dementia group requiring cautions for managing medicine	KDSQ_Score (dementia), K-MMSE_Score (dementia), SIADL_Finances (normal), SNSB_Stroop Test (normal), SIADL_Medications (dementia), K-MMSE_Day (dementia)	213 (70%)
7		A group in need of help for physical activities as they suffer some disability for movement and behaviors	KDSQ_Score (dementia), K-MMSE_Score (normal), SIADL_Finances (dementia), KDSQ_Difficulty in changing dirty clothes (dementia), SIADL_Transportation (dementia)	228 (77%)
8	A group showing false negative from initial screening examination, deterioration and difficulties in daily life	An initial dementia group	KDSQ_Score (normal), K-MMSE_Score (dementia), SNSB_Stroop Test (dementia), SIADL_Finances (dementia)	728 (83%)
9		Precise examination for depression is required and this is an initial dementia group	KDSQ_Score (normal), K-MMSE_Score (dementia), SNSB_Stroop Test (dementia), K-MMSE_Score (dementia), SIADL_Finances (dementia), SIADL_Leisure/hobbies (dementia),	262 (77%)
10	A group showing serious disorder especially for daily life although patients in this group suffer from deterioration of cognitive skills		KDSQ_Score (normal), K-MMSE_Score (normal), SIADL_Going out (dementia), SIADL_Finances (dementia), K-MMSE_Score (normal), SIADL_Shopping (dementia)	77 (77%)

**Fig. 6:**
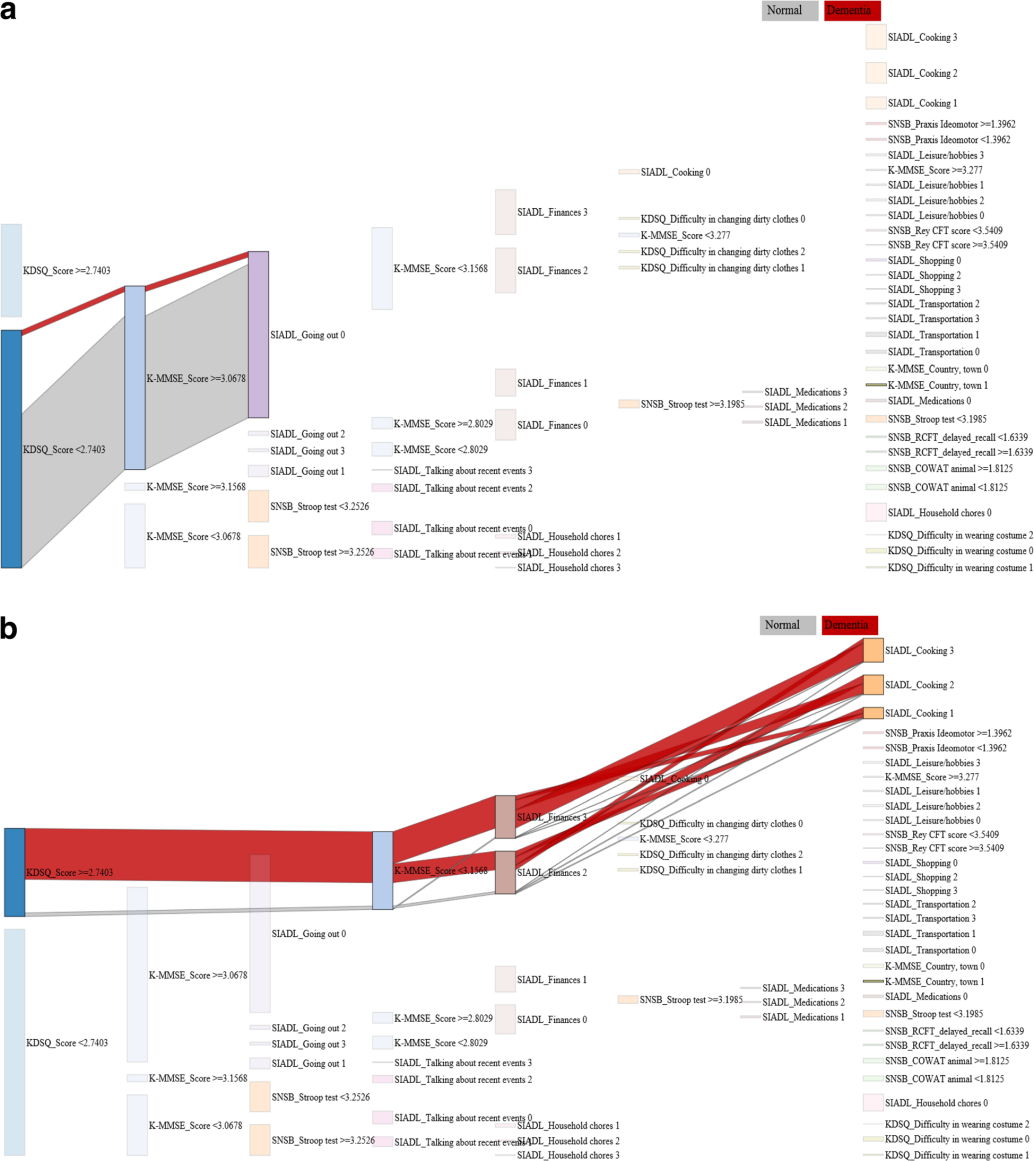
The results of visualization: **a** group1, and **b** group 3

We visualized 31 patient groups and visualization for all groups, refer to URL http://202.30.24.167:3000/ [35]. Visualization makes it effective to explore diagnostic reasoning of each groups by following the significant criteria from the left to the right side in the graph as do the DT. Also, people who predicted as normal or dementia expressed as green color and red color respectively, and different detailed diagnostic criteria are marked by colors. And thickness of lines is proportional to number of patients in each group. Lastly, this graph has made it possible to identify which criteria is more effective to determine a patient with dementia by laying highly-likely-diagnostic criteria for dementia on upper side of the graph and more-likely-to-be normal patient on bottom side of it. Two visualization results of group 1, group 3 are shown in Fig. 6 as typical cases. As shown in Fig. 6a, people included in group 1 have evaluation results of low KDSQ score, high K-MMSE score and no difficulty with going out by oneself. Therefore, almost of them are no deterioration of cognitive skills or with very weak symptom. On the other hand, people in group 3 in Fig. 6b have high KDSQ score, low K-MMSE score and answer 2 or 3 for SIADL related to finances and cooking. We can easily figure out that almost people in the group are with dementia symptom more than medium level.

## Conclusion

In this study, we proposed a data mining modeling for dementia diagnosis by analyzing a clinical data. The proposed model identifies needs for stepwise diagnostic process and suggests proper solution. The proposed quad-phased data mining modeling consists of proposer, predictor, descriptor, and visualization module. First of all, 48 diagnostic criteria which is 10 times of reduction compared to conventional things are suggested using a flexible variable selection method. And SVM with high performance of AUC 0.96 was configured as a subsidiary decision making model for a clinician. Finally for better understanding of predicted results, not only detailed profiles for 10 patient groups, but also visualization for a total 31 groups are provided.

Therefore, the data mining modeling is an intelligent system enabling intuitive collaboration between CAD system and physicians. Although various conventional studies have been trying to develop new system to diagnose dementia for last several decades and even until now, it was insufficient to be applied to the real clinical arena. However, The method proposed by this study have very meaningful clinic aspect with lots of possibilities to provide subsidiary information based on big data reflecting characteristics of patients with its new and different approach from conventional dementia researches. And also, improved evaluation process is able to effectively reduce time and cost consuming for clinicians and patients.

In the future, this study is believed to extract results to help actual treatment by classifying types of dementia on more detailed basis and identifying nature of dementia. Moreover, it would be able to acquire more meaningful clinical aspects by integrating brain image and information about dielectric substance and transcriptome, lastly it is required to study reconstructing of existing machine learning algorism to reflect unique characteristics of clinical data.

## Additional file

Additional file 1: Table of Contents. Table A: The list of selected variables from proposer module. Table B: The list of patient groups from descriptor module. (DOCX 35 kb)
