# Supplementary material for: Quad-phased data mining modeling for dementia diagnosis
Source: BMC Med Inform Decis Mak. 2017 May 18;17(Suppl 1):60. doi: 10.1186/s12911-017-0451-3 (PMC5444044; doi:10.1186/s12911-017-0451-3)
Supplement: Additional file 1: — Table of Contents. Table A: The list of selected variables from proposer module. Table B: The list of patient groups from descriptor module. (DOCX 35 kb) [file 12911_2017_451_MOESM1_ESM.docx]

**Table of Contents**

Table A: The list of selected variables from proposer module

Table B: The list of patient groups from descriptor module

**Table A.** The list of selected variables from proposer module

|  | **Categories** | **Variable Description** | **Data Type** | **k** |
| --- | --- | --- | --- | --- |
| 1 | Seoul-Instrumental Activities of Daily Living (S-IADL) | Using the telephone | Nominal | 6 |
| 2 |  | Shopping | Nominal | 4 |
| 3 |  | Preparing food/cooking | Nominal | 4 |
| 4 |  | Household chores | Nominal | 6 |
| 5 |  | Using transportation | Nominal | 6 |
| 6 |  | Going out (short distance) | Nominal | 6 |
| 7 |  | Taking medications | Nominal | 4 |
| 8 |  | Managing finances | Nominal | 4 |
| 9 |  | Grooming | Nominal | 6 |
| 10 |  | Keeping appointments | Nominal | 4 |
| 11 |  | Talking about recent events | Nominal | 4 |
| 12 |  | Leisure/hobbies | Nominal | 6 |
| 13 | Korean Dementia Screening Questionnaire  (KDSQ) | Does not know the date or day | Nominal | 6 |
| 14 |  | Does not wear outfits that fit time, place or occasion | Nominal | 4 |
| 15 |  | Finds it hard to go somewhere on his/her own using public transportation (except in the case of physical disability, such as arthritis) | Nominal | 6 |
| 16 |  | Refuses to change dirty underwear or clothes | Nominal | 4 |
| 17 |  | Score | Continuous | 6 |
| 18 | Basic Activity of Daily Living (BADL) | Personal hygiene and grooming | Nominal | 5 |
| 19 |  | Dressing | Nominal | 4 |
| 20 |  | Bathing | Nominal | 6 |
| 21 | Neuropsychiatric Inventory (NPI) | Delusion | Nominal | 5 |
| 22 |  | Distress(Delusion) | Nominal | 4 |
| 23 |  | Distress | Nominal | 4 |
| 24 |  | Total(Frequency) | Continuous | 4 |
| 25 |  | Total(severity) | Continuous | 4 |
| 26 | Demographic and baseline characteristics | Education | Nominal | 4 |
| 27 |  | Education(year) | Continuous | 6 |
| 28 | Korean Mini-Mental State Examination (K-MMSE) | What is the year? | Nominal | 6 |
| 29 |  | What is the season? | Nominal | 6 |
| 30 |  | What is the date? | Nominal | 6 |
| 31 |  | What is the day | Nominal | 6 |
| 32 |  | What is the month? | Nominal | 6 |
| 33 |  | Where are we state? | Nominal | 4 |
| 34 |  | Where are we country and town? | Nominal | 6 |
| 35 |  | Where are we hospital and floor? | Nominal | 6 |
| 36 |  | K-MMSE total score | Continuous | 6 |
| 37 | Seoul Neuropsychologic Screening Battery (SNSB) | Naming Adjusted S K BNT(Boston Naming Test) score | Continuous | 4 |
| 38 |  | Praxis Ideomotor | Continuous | 6 |
| 39 |  | Rey CFT(Complex Figure Test) copy score | Continuous | 6 |
| 40 |  | SVLT(Seoul Verbal Learing Test ) recall trial3 | Continuous | 6 |
| 41 |  | SVLT recall total score | Continuous | 4 |
| 42 |  | SVLT Delayed recall | Continuous | 4 |
| 43 |  | SVLT recognition score | Continuous | 5 |
| 44 |  | SVLT recognition discriminability index | Continuous | 6 |
| 45 |  | Go No Go | Continuous | 5 |
| 46 |  | COWAT Controlled Oral Word Association Test animal | Continuous | 6 |
| 47 |  | COWAT Controlled Oral Word Association Test supermarket | Continuous | 6 |
| 48 |  | Stroop Test Colorreading correct | Continuous | 4 |
| 49 | [Target] binary CDR |  | Binary |  |

**Table B.** The list of patient groups from descriptor module

| **id** | **Tree depth 1** | **Tree depth 2** | **Tree depth 3** | **Tree depth 4** | **Tree depth 5** | **Tree depth 6** |
| --- | --- | --- | --- | --- | --- | --- |
| 1 | KDSQ_Score | K-MMSE_Score | SIADL_Going out |  |  |  |
|  | < 2.7403 | >=3.0678 | 0 or missing |  |  |  |
| 2 | KDSQ_Score | K-MMSE_Score | SIADL_Going out | SIADL_Finances |  |  |
|  | < 2.7403 | >=3.0678 | 1,2,3 | 0,1 or missing |  |  |
| 3 | KDSQ_Score | K-MMSE_Score | SIADL_Finances | SNSB_Stroop Test |  |  |
|  | >=2.7403 | <3.1568 | 0,1 | <3.1985 |  |  |
| 4 | KDSQ_Score | K-MMSE_Score | SIADL_Finances | SIADL_Cooking |  |  |
|  | >=2.7403 | <3.1568 | 2,3 | 1,2,3 |  |  |
| 5 | KDSQ_Score | K-MMSE_Score | SIADL_Finances | SIADL_Going out |  |  |
|  | >=2.7403 | >=3.1568 | 0,1 | 0 or missing |  |  |
| 6 | KDSQ_Score | K-MMSE_Score | SNSB_Stroop Test | K-MMSE_Score | SIADL_Finances |  |
|  | < 2.7403 | <3.0678 | <3.2526 | <2.8029 | 0,2,3 |  |
| 7 | KDSQ_Score | K-MMSE_Score | SNSB_Stroop Test | K-MMSE_Score | SIADL_Finances |  |
|  | < 2.7403 | <3.0678 | <3.2526 | <2.8029 | 0,2,3 |  |
| 8 | KDSQ_Score | K-MMSE_Score | SNSB_Stroop Test | SIADL_Talking about recent events | SIADL_Household chores |  |
|  | < 2.7403 | <3.0678 | >=3.2526 or missing | 0,1 or missing | 0 or missing |  |
| 9 | KDSQ_Score | K-MMSE_Score | SNSB_Stroop Test | SIADL_Talking about recent events | SIADL_Finances |  |
|  | < 2.7403 | <3.0678 | >=3.2526 or missing | 2,3 | 2,3 |  |
| 10 | KDSQ_Score | K-MMSE_Score | SIADL_Going out | SIADL_Finances | K-MMSE_Score |  |
|  | < 2.7403 | >=3.0678 | 1,2,3 | 2,3 | >=3.277 |  |
| 11 | KDSQ_Score | K-MMSE_Score | SIADL_Finances | SNSB_Stroop Test | SIADL_Medications |  |
|  | >=2.7403 | <3.1568 | 0,1 | >=3.1985 | 0 |  |
| 12 | KDSQ_Score | K-MMSE_Score | SIADL_Finances | SIADL_Cooking | SNSB_Praxis Ideomotor |  |
|  | >=2.7403 | <3.1568 | 2,3 | 0 | <1.3962 |  |
| 13 | KDSQ_Score | K-MMSE_Score | SIADL_Finances | SIADL_Cooking | SNSB_Praxis Ideomotor |  |
|  | >=2.7403 | <3.1568 | 2,3 | 0 | >=1.3962 |  |
| 14 | KDSQ_Score | K-MMSE_Score | SIADL_Finances | KDSQ_Difficulty in changing dirty clothes | SIADL_Transportation |  |
|  | >=2.7403 | >=3.1568 | 2,3 | 1,2 or missing | 1,2,3 or missing |  |
| 15 | KDSQ_Score | K-MMSE_Score | SIADL_Finances | KDSQ_Difficulty in changing dirty clothes | SIADL_Transportation |  |
|  | >=2.7403 | >=3.1568 | 2,3 | 1,2 or missing | 0 |  |
| 16 | KDSQ_Score | K-MMSE_Score | SIADL_Finances | KDSQ_Difficulty in changing dirty clothes | SNSB_Rey CFT score |  |
|  | >=2.7403 | >=3.1568 | 2,3 | 0 | <3.5409 |  |
| 17 | KDSQ_Score | K-MMSE_Score | SIADL_Finances | KDSQ_Difficulty in changing dirty clothes | SNSB_Rey CFT score |  |
|  | >=2.7403 | >=3.1568 | 2,3 | 0 | >=3.5409 |  |
| 18 | KDSQ_Score | K-MMSE_Score | SIADL_Finances | SIADL_Going out | SNSB_RCFT_delayed_recall |  |
|  | >=2.7403 | >=3.1568 | 0,1 or missing | 1,2,3 | <1.6339 or missing |  |
| 19 | KDSQ_Score | K-MMSE_Score | SIADL_Finances | SIADL_Going out | SNSB_RCFT_delayed_recall |  |
|  | >=2.7403 | >=3.1568 | 0,1 or missing | 1,2,3 | >=1.6339 |  |
| 20 | KDSQ_Score | K-MMSE_Score | SNSB_Stroop Test | K-MMSE_Score | SIADL_Finances | SIADL_Leisure/hobbies |
|  | < 2.7403 | <3.0678 | <3.2526 | >=2.8029 | 2,3 | 2,3 or missing |
| 21 | KDSQ_Score | K-MMSE_Score | SNSB_Stroop Test | K-MMSE_Score | SIADL_Finances | SIADL_Leisure/hobbies |
|  | < 2.7403 | <3.0678 | <3.2526 | >=2.8029 | 2,3 | 0,1 |
| 22 | KDSQ_Score | K-MMSE_Score | SNSB_Stroop Test | K-MMSE_Score | SIADL_Finances | SNSB_COWAT supermarket |
|  | < 2.7403 | <3.0678 | <3.2526 | >=2.8029 | 0,1 or missing | <1.8125 |
| 23 | KDSQ_Score | K-MMSE_Score | SNSB_Stroop Test | K-MMSE_Score | SIADL_Finances | SNSB_COWAT supermarket |
|  | < 2.7403 | <3.0678 | <3.2526 | >=2.8029 | 0,1 or missing | >=1.8125 |
| 24 | KDSQ_Score | K-MMSE_Score | SNSB_Stroop Test | SIADL_Talking about recent events | SIADL_Household chores | KDSQ_Difficulty in wearing costume |
|  | < 2.7403 | <3.0678 | >=3.2526 or missing | 0,1 or missing | 1,2,3 | 0,2 or missing |
| 25 | KDSQ_Score | K-MMSE_Score | SNSB_Stroop Test | SIADL_Talking about recent events | SIADL_Household chores | KDSQ_Difficulty in wearing costume |
|  | < 2.7403 | <3.0678 | >=3.2526 or missing | 0,1 or missing | 1,2,3 | 1 |
| 26 | KDSQ_Score | K-MMSE_Score | SNSB_Stroop Test | SIADL_Talking about recent events | SIADL_Finances | SIADL_Transportation |
|  | < 2.7403 | <3.0678 | >=3.2526 or missing | 2,3 | 0.1 or missing | 0 or missing |
| 27 | KDSQ_Score | K-MMSE_Score | SNSB_Stroop Test | SIADL_Talking about recent events | SIADL_Finances | SIADL_Transportation |
|  | < 2.7403 | <3.0678 | >=3.2526 or missing | 2,3 | 0.1 or missing | 1,2,3 |
| 28 | KDSQ_Score | K-MMSE_Score | SIADL_Going out | SIADL_Finances | K-MMSE_Score | SIADL_Shopping |
|  | < 2.7403 | >=3.0678 | 1,2,3 | 2,3 | <3.277 | 2,3 |
| 29 | KDSQ_Score | K-MMSE_Score | SIADL_Going out | SIADL_Finances | K-MMSE_Score | SIADL_Shopping |
|  | < 2.7403 | >=3.0678 | 1,2,3 | 2,3 | <3.277 | 0,1 or missing |
| 30 | KDSQ_Score | K-MMSE_Score | SIADL_Finances | SNSB_Stroop Test | SIADL_Medications | K-MMSE_Day |
|  | >=2.7403 | <3.1568 | 0,1 | >=3.1985 | 1,2,3 or missing | 1 |
| 31 | KDSQ_Score | K-MMSE_Score | SIADL_Finances | SNSB_Stroop Test | SIADL_Medications | K-MMSE_Day |
|  | >=2.7403 | <3.1568 | 0,1 | >=3.1985 | 1,2,3 or missing | 0 or missing |
